# Stuttering Priapism in a Dog—First Report

**DOI:** 10.3390/vetsci9100518

**Published:** 2022-09-23

**Authors:** Françoise A. Roux, Florian Le Breuil, Julien Branchereau, Jack-Yves Deschamps

**Affiliations:** 1 Emergency and Critical Care Unit, Oniris, Nantes-Atlantic College of Veterinary Medicine, Food Science and Engineering, La Chantrerie, CS 40706, CEDEX 03, 44307 Nantes, France; 2 Nutrition, PathoPhysiology and Pharmacology (NP3) Unit, Oniris, Nantes-Atlantic College of Veterinary Medicine, Food Science and Engineering, La Chantrerie, CS 40706, CEDEX 03, 44307 Nantes, France; 3 Institut de Transplantation-Urologie-Néphrologie, CHU de Nantes, 30 boulevard Jean Monnet, CEDEX 01, 44093 Nantes, France

**Keywords:** priapism, stuttering priapism, recurrent priapism, intermittent priapism, ischemic priapism, erection disorder, erectile dysfunction, penis, dog

## Abstract

**Simple Summary:**

A 5-year-old recently castrated male Doberman dog presented for prolonged erection of one week’s duration with associated pain and dysuria. This was the fourth episode within a year. Each episode was associated with an unusual event, which was stressful for the dog. Castration performed two months prior to the final episode did not prevent recurrence. Due to tissue necrosis, penile amputation and urethrostomy had to be performed. To our knowledge, this case is the first report of a stuttering priapism in a dog. Stuttering priapism, also called recurrent or intermittent priapism, is a particular type of ischemic priapism described in humans that is characterized by repeated episodes of prolonged erections.

**Abstract:**

A 5-year-old recently castrated male Doberman dog presented for prolonged erection of one week’s duration with associated pain and dysuria. This was the fourth episode within a year. Each episode was associated with an unusual event, which was stressful for the dog. Castration performed two months prior to the final episode did not prevent recurrence. Due to tissue necrosis, penile amputation and urethrostomy had to be performed. The dog recovered fully. Prolonged erection that persists beyond or that is unrelated to sexual stimulation is called “priapism”. This term refers to the Greek god Priapus, a god of fertility, memorialized in sculptures for his giant phallus. In humans, depending on the mechanism involved, priapism is classified as nonischemic or ischemic. Because prognosis and treatment are different, priapism must be determined to be nonischemic or ischemic. Nonischemic priapism is a rare condition observed when an increase in penile arterial blood flow overwhelms the capacity of venous drainage; it is often associated with penile trauma, and does not require medical intervention. Ischemic priapism is associated with decreased venous return. In humans, ischemic priapism accounts for 95% of cases, the majority of which are idiopathic. Ischemic priapism is a urological emergency; simple conservative measures such as aspiration of blood from the corpora cavernosa and intracavernosal injection of an adrenergic agent are often successful. Stuttering priapism, also called recurrent or intermittent priapism, is a particular form of ischemic priapism reported in humans that is characterized by repetitive episodes of prolonged erections. Management consists of treating each new episode as an episode of acute ischemic priapism, and preventing recurrence with oral medications such as dutasteride and/or baclofen, gabapentin, or tadalafil. To the authors’ knowledge, this case is the first report of stuttering priapism in a dog.

## 1. Introduction

Priapism is a prolonged erection that persists beyond or is unrelated to sexual stimulation [1,2]. The term “priapism” refers to the Greek god Priapus, a god of fertility, memorialized in sculptures for his giant phallus. Priapism results from the absence of detumescence after erection.

Priapism may easily be confused with paraphimosis, a condition in which the non-erect penis cannot be reduced into the prepuce [3,4]. Paraphimosis may occur when the preputial orifice is too small, when the length of the prepuce is inadequate, when preputial muscles are weakened, or after a trauma [5]. Paraphimosis can lead to penile edema mimicking an erection, hence the possible confusion with priapism; priapism and paraphimosis can coexist [5].

The penis has three erectile body types: the glans, the corpus spongiosum, and the corpora cavernosa; priapism results from an engorgement of the corpora cavernosa because of an increase in arterial inflow or a decrease in venous outflow. The glans and the corpus spongiosum are mostly spared. Penile erection is a complex neurovascular event involving the central nervous system, the autonomic nervous system, and the vascular tissue of the penis. Penile erection and penile detumescence are controlled by the autonomic nervous system [6]. Penile erection is mediated through the pelvic nerve, composed of parasympathetic fibers. Parasympathetic stimulation of the pelvic nerve leads to penile arterial dilatation that increases arterial blood flow to the corpora cavernosa; subsequently, venous outflow decreases via contraction of smooth muscle fibers of the corpora cavernosa; this results in arterial engorgement. Penile detumescence occurs after sympathetic stimulus. Adrenergic sympathetic nerves release norepinephrine that causes relaxation of smooth muscle fibers, allowing drainage via the pudendal veins. Priapism is a persistent erection caused by disturbances in the mechanisms controlling penile detumescence [7]; the erection can appear de novo (as a result of a prolonged nocturnal erection, for instance) or may persist after sexual intercourse or masturbation [7]. In humans, prolonged corpora cavernosa ischemia lasting more than 24 to 48 h can lead to irreversible fibrosis with destruction of the erectile tissue, and subsequently to permanent erectile dysfunction in as many as 59% of cases [7]. In humans, priapism can be secondary to factors affecting penile erection like hematologic disease, trauma, surgery, neoplasia, neurological disorder, medication, or local inflammation [7]. Priapism is estimated to be idiopathic in 50% to 60% of cases [7].

Priapism has been reported in humans, in horses [8,9,10,11,12,13], in dogs, and in cats [14,15,16,17]. In all species, priapism is uncommon. We found 14 reports of priapism in dogs in the veterinary literature (excluding cases published in textbooks). Causes were trauma during copulation [14,18], neurological disorder (encephalomyelitis [19], disc herniation [5], syringohydromyelia [5], lumbar stenosis [20]), perineal disease [21,22], penile metastasis of a prostatic sarcoma [23] and unidentified causes [3,5,22,24]. The present case explores an unusual clinical presentation of priapism in a dog.

## 2. Case Report

A 5-year-old recently castrated male Doberman dog presented for lethargy, dysuria, stranguria and enlargement of the penis of seven days’ duration. The dog was very anxious. The owners reported three previous acute episodes of enlargement of the penis associated with dysuria and pain within a year prior to castration. In each case, reintegration of the penis into the prepuce was possible. According to the owners, these episodes were always associated with an unusual event, which was stressful for the dog, such as the absence or illness of the owner. During the first episode one year previously, radiographic and ultrasonographic examination of the urogenital tract did not reveal any abnormality, although penile ultrasound with color flow Doppler was not performed. Clinical signs resolved within 10 days. The dog, then intact, was given delmadinone acetate. The other two episodes occurred six months and two months prior to presentation. Each time, temporary urethral catheterization was performed; cephalexin and prednisolone were prescribed by the veterinarian. Clinical signs resolved within three to five days. The dog was neutered two months prior to the fourth episode. The fourth episode began seven days prior to presentation, while the dog was particularly anxious because the owner was away. The treatment used in previous episodes was tried, without effect. After seven days, because detumescence had not occurred, the dog was referred to the emergency unit of our institution.

**Figure 1:**
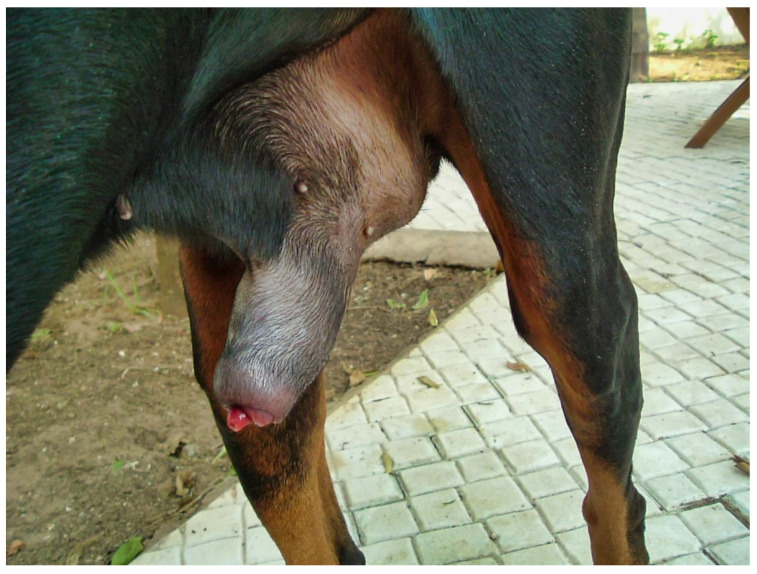
Marked enlargement of the penis, which protrudes slightly from the prepuce.

The day of presentation, physical examination was normal except for a tachypnea, which was probably due to pain. Genital examination showed a marked engorgement of the penis, which protruded slightly from the prepuce (Figure 1).

**Figure 2:**
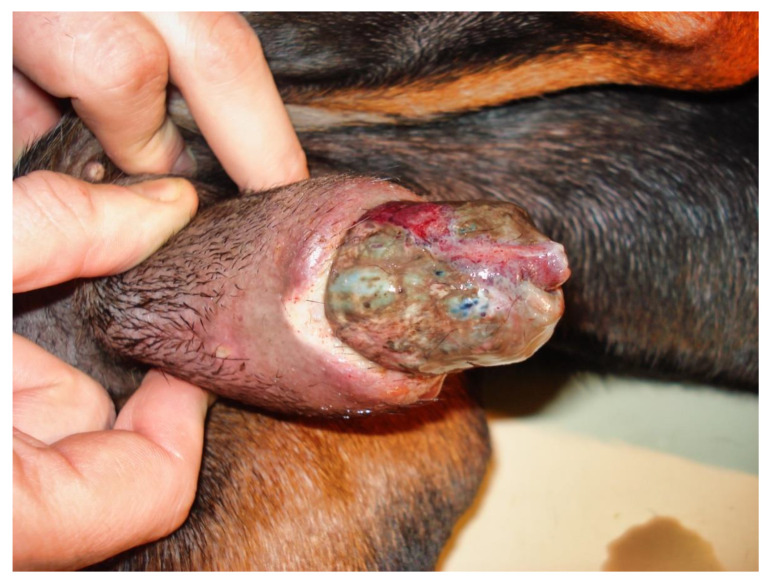
Partial exteriorization of the penis revealing a markedly engorged, rigid penis, dark purple in color with a darker area.

Partial exteriorization of the penis revealed a markedly engorged, rigid penis, dark purple in color with a darker area (Figure 2).

The penis was cold and extremely painful upon palpation. A pulse was not palpable on the penis. Neurologic and orthopedic examinations were normal. The diagnosis was priapism associated with partial necrosis of the penis and partial urethral obstruction. Complete blood count, coagulation status, and chemistry were normal. Radiographs of the urogenital tract confirmed enlargement of the penis and distension of the bladder. Ultrasonography showed engorged penile veins around the bulbus glandis of the penis. A penile doppler ultrasound exam did not show blood flow. Urethral catheterization produced brown-colored urine; no bacteria were found on microscopic examination.

**Figure 3:**
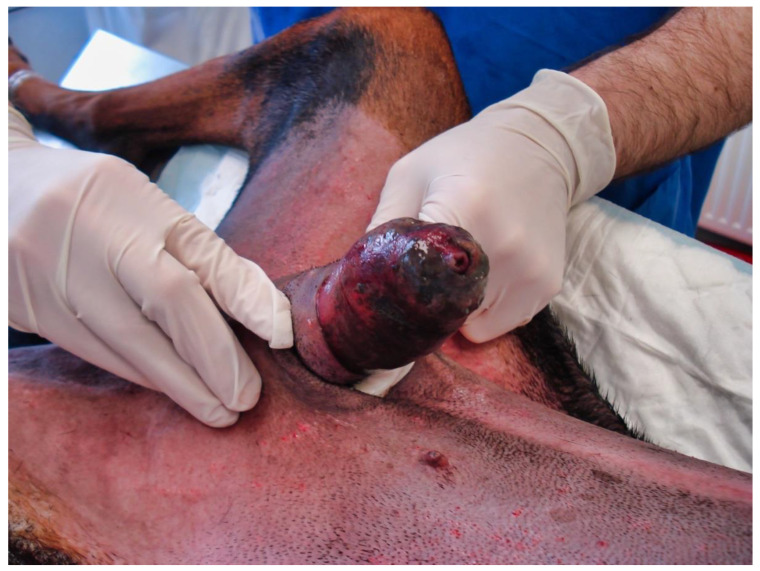
Extent of penile necrosis the day following admission.

**Figure 4:**
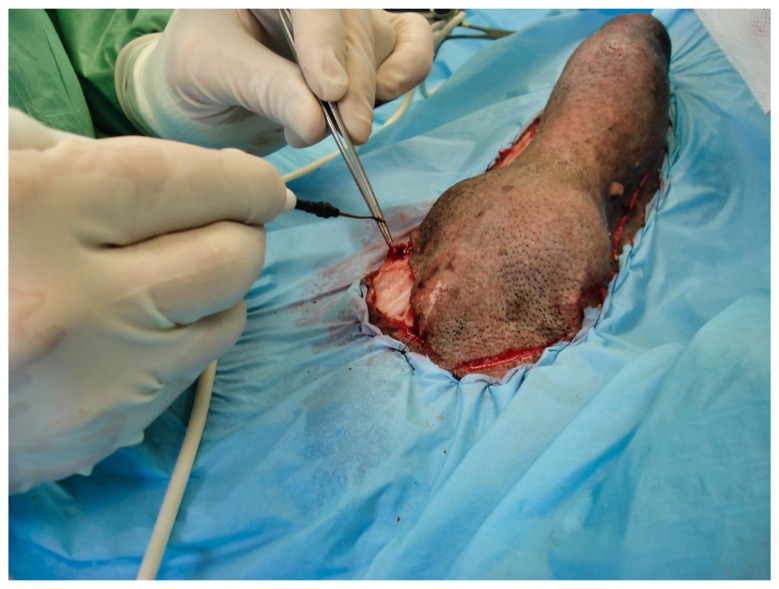
Intraoperative view of penile amputation, showing the limits of the incision.

**Figure 5:**
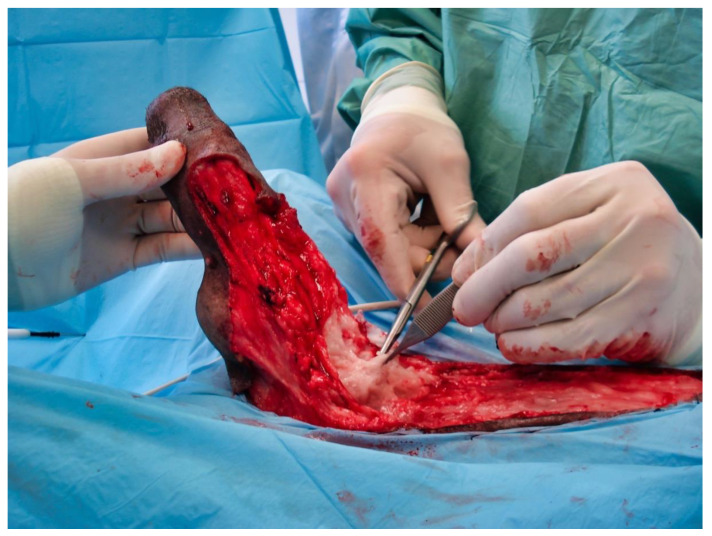
Intraoperative view of the amputation of the penis.

**Figure 6:**
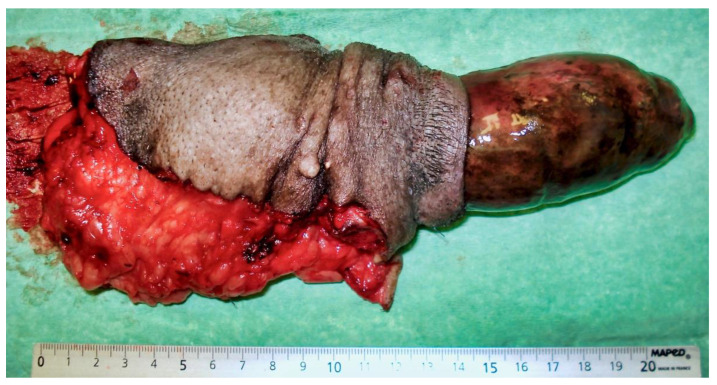
Resected specimen with the sheath still partially covering the penis.

**Figure 7:**
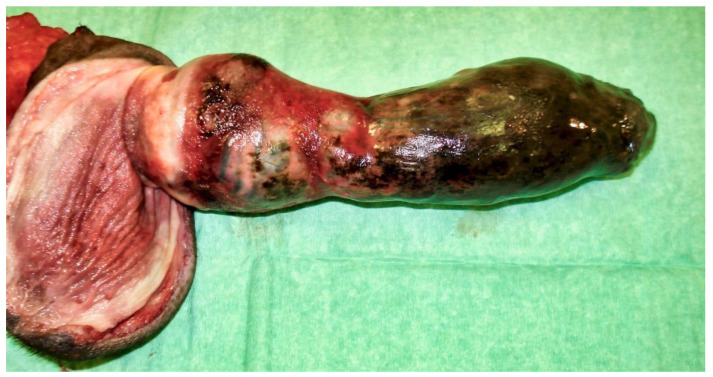
Resected specimen showing a more marked necrosis at the tip of the penis.

**Figure 8:**
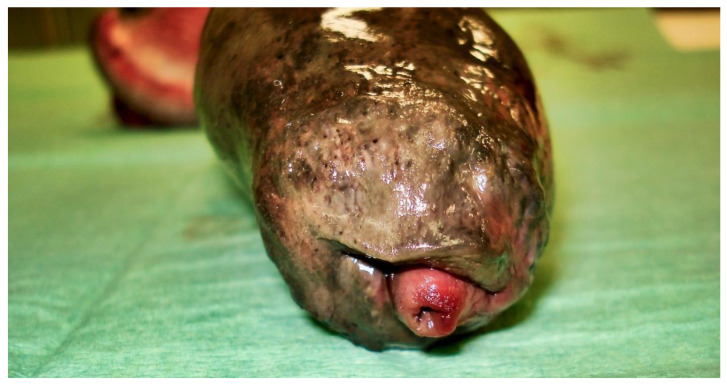
Necrosis at the tip of the penis.

**Figure 9:**
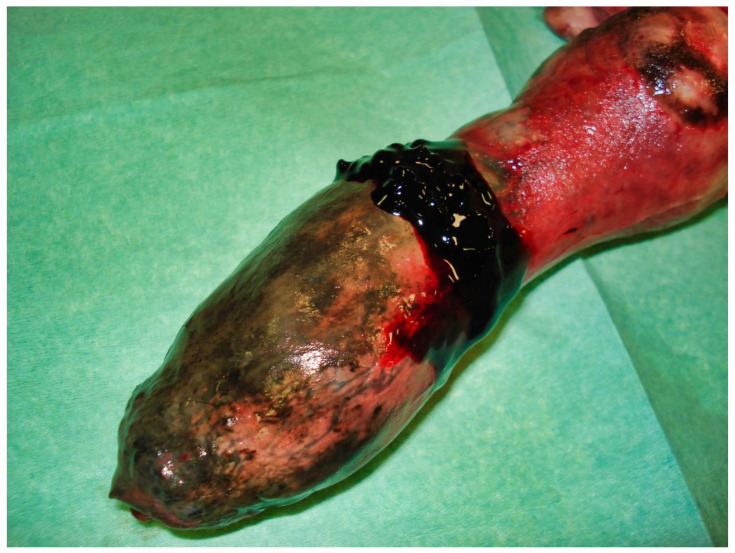
Black coagulum observed after incision of a necrotic area of the penis.

**Figure 10:**
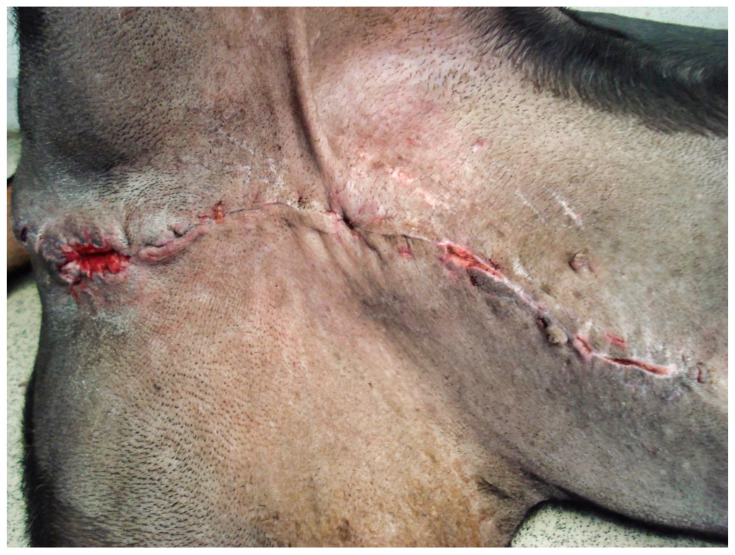
Urethrostomy wound and skin wound the tenth day following the surgery.

The following day, despite the application of ice packs to the sheath, because of the extent of penile necrosis (Figure 3), and the worsening of the dysuria, surgical treatment was elected (Figure 4), consisting of complete amputation of the penis (Figure 5) and scrotal urethrostomy. Examination of the resected specimen revealed necrosis of a large part of the penis (Figure 6), especially at its tip (Figure 7 and Figure 8). An incision into a necrotic area produced a black coagulum (Figure 9). There were no intraoperative or postoperative complications. The dog was discharged five days after presentation; he recovered fully (Figure 10). Histopathological examination confirmed a diffuse necrosis of the penis associated with multiple hemorrhages totally disrupting normal tissue architecture; no neoplastic process was observed. Except for the association with a stressful event, the cause of the priapism has not been precisely identified. The final diagnosis was idiopathic “recurrent priapism”, also called “stuttering priapism.”

## 3. Discussion

Advances in erection physiology in humans have led to a better understanding of the pathophysiology of priapism and its management. The goal of therapy of priapism in humans is to maintain erectile function. Priapism results from engorgement of the corpora cavernosa due to an increase in arterial inflow or a decrease in venous outflow. In humans, depending on the mechanism involved, priapism is classified as nonischemic or ischemic [1,25,26,27]. Treatment and prognosis differ accordingly. Given the similarity of pathophysiology, an algorithm identical to that of humans is reasonable for dogs [5].

### 3.1. Nonischemic Priapism

Nonischemic (arterial, high-flow) priapism is an uncommon form of persistent erection observed when an increase in arterial blood flow overwhelms the capacity of venous drainage [1,2]. The most common identified cause is trauma to the penis or to the perineum with traumatic rupture of the cavernous artery or its branches. Usually, the penis is non-rigid and non-painful and penile trauma is reported. Time from trauma to presentation has no significant impact on outcome. Nonischemic priapism is not an emergency: it often resolves without treatment. Nonischemic priapism has been reported in dogs [5,18] and in a cat [17]; as in humans, nonpainful, nonischemic priapism generally had a favorable outcome in dog and in cat.

### 3.2. Ischemic Priapism

Ischemic (veno-occlusive, low-flow) priapism is associated with a decrease in venous return. In humans, ischemic priapism accounts for 95% of cases [27,28]; the majority of cases are idiopathic [28]; some cases are associated with medications [29]. Usually, the penis is rigid and very painful. A penile doppler ultrasound exam does not show blood flow [30]. As in humans, most cases reported in dogs are ischemic priapisms. In the present case, the ischemic nature of the priapism is attested by the degree of pain, the rigidity of the penis, the absence of palpable pulsation, the absence of blood flow on doppler ultrasound exam, the extent of necrosis, and the black color of the blood within the corpora cavernosa.

Ischemic priapism requires emergency treatment; the outcome is time-dependent. The goal of therapy is to decompress the corpora cavernosa and restore blood flow. The American Urology Association (AUA), the European Association of Urology (EAU), and the Sexual Medicine Society of North America (SMSNA) have published guidelines on the management of ischemic priapism in humans [1,31,32]. Three recent reviews have also been published [33,34,35]. Guidelines recommend a stepwise escalation of treatment beginning with conservative treatment before invasive treatment [7].

#### 3.2.1. Aspiration

A stepwise approach to ischemic priapism starts with corporeal aspiration of blood with or without irrigation with non-heparinized saline solution [7]. The aspiration should be performed with an 18- or a 19-gauge needle placed at the base of the penis in the 3 o’clock and 9 o’clock positions [33]. The decompression of the corpora cavernosa by aspiration should be performed until detumescence is achieved, or fresh red blood is obtained. Aspiration is both therapeutic and diagnostic: cavernosal blood in men with ischemic priapism has pH < 7.25, PO_2_ < 30 mmHg and PCO_2_ > 60 mmHg; in men with nonischemic priapism, cavernosal blood values are similar to those of arterial blood. Aspiration provides pain relief. Aspiration +/− irrigation is about 30% successful in people [5].

#### 3.2.2. Intracavernous Injection of Sympathomimetic Agents

If ischemic priapism persists despite aspiration, intracavernous injection of a sympathomimetic drug should be performed; the panel recommends use of diluted phenylephrine because of its limited cardiovascular risks compared with drugs with β-adrenergic activity. Other α-adrenergic agonists such as ephedrine, epinephrine or norepinephrine may be used [33]; there are no published data that compare the efficacity of the different drugs [33]. The phenylephrine is diluted in normal saline solution to 100 to 500 μg/mL; 100 to 200 μg every 5 min is injected until detumescence is achieved, with a maximal dose of 1000 μg (1 mg) in an adult human [5,33]. As no blood is retained in the penis, to avoid systemic complications, it is important to inject the phenylephrine into the corporeal cavernosa only and the patient should be monitored closely during the injection and for an hour after the injection [33]. Appropriate dosages have not been determined in dogs and cats; Lavely suggests starting with a low dosage (1–3 μg/kg) [5]. Intracavernous injection can cause pain [7], so it is preferable to perform it with the patient under sedation or anesthesia [5]. In humans, the resolution rate with aspiration and intracavernous injection of sympathomimetic agents ranges from 43% to 81% [5].

It is regrettable that in the present case, neither aspiration nor intracavernous injection of an α-adrenergic agonist was attempted on admission. This could have prevented the worsening of penile necrosis and avoided amputation surgery. The clotted blood removed after amputation (Figure 9) is typical of that removed by aspiration in humans, and suggests that aspiration could have been beneficial. This article is a reminder of these simple, little-known measures, since priapism in dogs is so rare. Among the simple measures to put in place, lubrication of the penis to limit tissue damage secondary to exposure and the use of an Elizabethan collar to prevent self-mutilation should not be neglected. Urethral catheterization is sometimes required.

#### 3.2.3. Surgical Intervention

In humans, in the case of failure of conservative options, surgery should be considered [28]. Shunt surgery diverts blood for the corpus cavernosa into another area. Penile prosthesis allows the recovery of sexual function in patients with erectile dysfunction. In a castrated dog, because erectile function is not expected, amputation of the penis is a reasonable option. Prolonged erection over 4 h induces local hypoxia and acidosis; this initiates a cascade of events leading to the formation of thrombi in the sinusoidal spaces and smooth muscle cell necrosis within 24 h [36]. In the present case, the dog presented seven days after the onset of priapism, with extensive necrosis of the penis; because of the worsening of the necrosis, penile amputation was required. In a case series of penile amputation and scrotal urethrostomy in 18 dogs, 4 dogs had priapism [22].

### 3.3. Stuttering Priapism

What make this case unique to the veterinary literature is that the dog had three previous episodes of priapism. These findings are consistent with stuttering priapism described in humans. Stuttering priapism, also called recurrent or intermittent priapism, is a particular type of ischemic priapism that is characterized by repetitive episodes of prolonged erections [1,30,31,37,38]. Typically, the episodes are painful, sleep-related, last less than 3 h and are self-limiting [39]. However, these episodes can increase in duration and in incidence, leading to acute major ischemic priapism episodes that require emergency medical management [33]; 28% of patients progress to major episodes of ischemic priapism [40] that can lead to irreversible corporal fibrosis with permanent erectile dysfunction. Management of stuttering priapism consists in preventing future episodes with oral medications, and in treating each new episode following the recommendations for acute ischemic priapism previously reported [41]. Because the mechanisms involved in the development of stuttering priapism are poorly characterized, medical management is often disappointing [42].

#### 3.3.1. Hormonal Therapy

Hormonal therapy has sometimes been a successful medical option for preventing recurrence of stuttering priapism in humans [42]. In a study including 13 patients, the 5-α reductase inhibitor dutasteride led to some degree of improvement in 11/13 (85%) men: 5/13 (38%) had complete resolution of their symptoms and 6/13 (46%) had reduced frequency and/or severity of their episodes [43]; in comparison to finasteride, which has a half-life of 5 h, dutasteride has a half-life of 35 days.

In the present case, castration two months prior to the fourth episode did not prevent the recurrence. According to the owners, the association between each episode of priapism and a stressful event was clear; given that the dog was sexually intact at the time of the first three episodes, it is not excluded that sexual stimulation such as masturbation was involved. In a case series including 21 human patients without identifiable cause for their priapism, an anxiety disorder was self-reported in 10 patients [44].

There are no clear guidelines on the prevention of stuttering priapism in humans; in dogs, considering the published data in humans, any testosterone abnormalities should be corrected; dutasteride can be used, but surgical castration is a reasonable option.

#### 3.3.2. Non Hormonal Therapies

There are anecdotical reports of the use of non-hormonal therapies with oral baclofen [45,46,47,48], oral terbutaline (a β2-agonist) [49], oral salbutamol (a β2-agonist) [50], oral gabapentin (an anticonvulsant drug) [51] and oral tadalafil (a phosphodiesterase type 5 (PDE5) inhibitor) [52,53,54].

## 4. Conclusions

Because prognosis and treatment are different, priapism must be determined to be ischemic or nonischemic. Nonischemic priapism is a rare condition, often associated with penile trauma, that does not require medical intervention. Conversely, ischemic priapism is a urological emergency; simple conservative measures like aspiration of the corpora cavernosa and intracavernosal injection of an adrenergic agent are often successful. Stuttering priapism, also called intermittent or recurrent priapism, is a particular form of ischemic priapism reporting in humans. Management in humans consists in treating each new episode as acute ischemic priapism, and preventing recurrence with oral medications like dutasteride and/or baclofen, gabapentin, or tadalafil. To the authors’ knowledge, this case is the first report of a stuttering priapism in a dog.

## Data Availability

All data are available on request.

## References

[1] Montague D.K., Jarow J., Broderick G.A., Dmochowski R.R., Heaton J.P., Lue T.F., Nehra A., Sharlip I.D., Members of the Erectile Dysfunction Guideline Update Panel, Americal Urological Association (2003). American Urological Association guideline on the management of priapism. J. Urol..

[2] Levey H.R., Segal R.L., Bivalacqua T.J. (2014). Management of priapism: An update for clinicians. Ther. Adv. Urol..

[3] Kustritz M.V., Olson P.N. (1999). Theriogenology question of the month. Priapism or paraphimosis. J. Am. Vet. Med. Assoc..

[4] Rochat M.C. (2001). Priapism: A review. Theriogenology.

[5] Lavely J.A. (2009). Priapism in dogs. Top Companion Anim. Med..

[6] Jung J., Jo H.W., Kwon H., Jeong N.Y. (2014). Clinical neuroanatomy and neurotransmitter-mediated regulation of penile erection. Int. Neurourol. J..

[7] Van der Horst C., Stuebinger H., Seif C., Melchior D., Martinez-Portillo F.J., Juenemann K.P. (2003). Priapism—Etiology, pathophysiology and management. Int. Braz. J. Urol..

[8] Pearson H., Weaver B.M. (1978). Priapism after sedation, neuroleptanalgesia and anaesthesia in the horse. Equine. Vet. J..

[9] Gerring E.L. (1981). Priapism and ACP in the horse. Vet. Rec..

[10] Schumacher J., Hardin D.K. (1987). Surgical treatment of priapism in a stallion. Vet. Surg..

[11] Blanchard T.L., Schumacher J., Edwards J.F., Varner D.D., Lewis R.D., Everett K., Joyce J.R. (1991). Priapism in a stallion with generalized malignant melanoma. J. Am. Vet. Med. Assoc..

[12] Wilson D.V., Nickels F.A., Williams M.A. (1991). Pharmacologic treatment of priapism in two horses. J. Am. Vet. Med. Assoc..

[13] Van Harreveld P.D., Gaughan E.M. (1999). Partial phallectomy to treat priapism in a horse. Aust. Vet. J..

[14] Orima H., Tsutsui T., Waki T., Kawakami E., Ogasa A. (1989). Surgical treatment of priapism observed in a dog and a cat. Nihon Juigaku Zasshi. Jpn. J. Vet. Sci..

[15] Swalec K.M., Smeak D.D. (1989). Priapism after castration in a cat. J. Am. Vet. Med. Assoc..

[16] Gunn-Moore D.A., Brown P.J., Holt P.E., Gruffydd-Jones T.J. (1995). Priapism in seven cats. J. Small Anim. Pract..

[17] Lee J.M., Sung A.W., Lee H.J., Song J.H., Song K.H. (2022). Presumptive Non-Ischemic Priapism in a Cat. Vet. Sci..

[18] El-Sherry T., Abdel-Ghani M. (2018). Non-ischemic priapism in dog: Case report. Asian Pac. J. Reprod..

[19] Guilford W.G., Shaw D.P., O’Brien D.P., Maxwell V.D. (1990). Fecal incontinence, urinary incontinence, and priapism associated with multifocal distemper encephalomyelitis in a dog. J. Am. Vet. Med. Assoc..

[20] Payan-Carreira R., Colaco B., Rocha C., Albuquerque C., Luis M., Abreu H., Pires M.A. (2013). Priapism associated with lumbar stenosis in a dog. Reprod. Domest. Anim..

[21] Martins-Bessa A., Santos T., Machado J., Pinelas R., Pires M.A., Payan-Carreira R. (2008). Priapism secondary to perineal abscess in a dog—A case report. Reprod Domest Anim..

[22] Burrow R.D., Gregory S.P., Giejda A.A., White R.N. (2011). Penile amputation and scrotal urethrostomy in 18 dogs. Vet. Rec..

[23] Rogers L., Lopez A., Gillis A. (2002). Priapism secondary to penile metastasis in a dog. Can. Vet. J..

[24] Park J., An S.A., Jeong S.M., Seo K.w. (2017). Idiopathic Ischemic Priapism in a Shih Tzu. J. Vet. Clin. Korean Soc. Vet. Clin..

[25] Burnett A.L., Bivalacqua T.J. (2007). Priapism: Current principles and practice. Urol. Clin. North Am..

[26] Bassett J., Rajfer J. (2010). Diagnostic and therapeutic options for the management of ischemic and nonischemic priapism. Rev. Urol..

[27] Broderick G.A., Kadioglu A., Bivalacqua T.J., Ghanem H., Nehra A., Shamloul R. (2010). Priapism: Pathogenesis, epidemiology, and management. J. Sex. Med..

[28] Ridgley J., Raison N., Sheikh M.I., Dasgupta P., Khan M.S., Ahmed K. (2017). Ischaemic priapism: A clinical review. Turk. J. Urol..

[29] Schifano N., Capogrosso P., Boeri L., Fallara G., Cakir O.O., Castiglione F., Alnajjar H.M., Muneer A., Deho F., Schifano F. (2022). Medications mostly associated with priapism events: Assessment of the 2015–2020 Food and Drug Administration (FDA) pharmacovigilance database entries. Int. J. Impot. Res..

[30] Liguori G., Rizzo M., Boschian R., Cai T., Palmieri A., Bucci S., Pavan N., Claps F., Boltri M., Bertolotto M. (2020). The management of stuttering priapism. Minerva Urol. Nefrol..

[31] Salonia A., Eardley I., Giuliano F., Hatzichristou D., Moncada I., Vardi Y., Wespes E., Hatzimouratidis K., European Association of Urology (2014). European Association of Urology guidelines on priapism. Eur. Urol..

[32] Bivalacqua T.J., Allen B.K., Brock G., Broderick G.A., Kohler T.S., Mulhall J.P., Oristaglio J., Rahimi L.L., Rogers Z.R., Terlecki R.P. (2021). Acute Ischemic Priapism: An AUA/SMSNA Guideline. J. Urol..

[33] Moussa M., Abou Chakra M., Papatsoris A., Dellis A., Peyromaure M., Barry Delongchamps N., Bailly H., Roux S., Yassine A.A., Duquesne I. (2022). An update on the management algorithms of priapism during the last decade. Arch. Ital. Urol. Androl..

[34] Biebel M.G., Gross M.S., Munarriz R. (2022). Review of Ischemic and Non-ischemic Priapism. Curr. Urol. Rep..

[35] Ericson C., Baird B., Broderick G.A. (2021). Management of Priapism: 2021 Update. Urol. Clin. North Am..

[36] Yuan J., Desouza R., Westney O.L., Wang R. (2008). Insights of priapism mechanism and rationale treatment for recurrent priapism. Asian J. Androl..

[37] Joice G.A., Liu J.L., Burnett A.L. (2021). Medical treatment of recurrent ischaemic priapism: A review of current molecular therapeutics and a new clinical management paradigm. BJU Int..

[38] Abdeen B.M., Leslie S.W. (2022). Stuttering Priapism. StatPearls.

[39] Morrison B.F., Burnett A.L. (2012). Stuttering priapism: Insights into pathogenesis and management. Curr. Urol. Rep..

[40] Emond A.M., Holman R., Hayes R.J., Serjeant G.R. (1980). Priapism and impotence in homozygous sickle cell disease. Arch. Intern. Med..

[41] Kheirandish P., Chinegwundoh F., Kulkarni S. (2011). Treating stuttering priapism. BJU Int..

[42] Levey H.R., Kutlu O., Bivalacqua T.J. (2012). Medical management of ischemic stuttering priapism: A contemporary review of the literature. Asian J. Androl..

[43] Baker R.C., Bergeson R.L., Yi Y.A., Ward E.E., Morey A.F. (2020). Dutasteride in the long-term management of stuttering priapism. Transl. Androl. Urol..

[44] Burnett A.L. (2009). Anxiety disorders in patients with idiopathic priapism: Risk factor and pathophysiologic link?. J. Sex. Med..

[45] Rourke K.F., Fischler A.H., Jordan G.H. (2002). Treatment of recurrent idiopathic priapism with oral baclofen. J. Urol..

[46] Vaidyanathan S., Watt J.W., Singh G., Hughes P.L., Selmi F., Oo T., Soni B.M., Sett P. (2004). Management of recurrent priapism in a cervical spinal cord injury patient with oral baclofen therapy. Spinal Cord..

[47] Moreira D.M., Pimentel M., da Silva Moreira B.F., Stein A.C., Koff W.J. (2006). Recurrent priapism in the young patient treated with baclofen. J. Pediatr. Urol..

[48] Johnson M.J., McNeillis V., Chiriaco G., Ralph D.J. (2021). Rare Disorders of Painful Erection: A Cohort Study of the Investigation and Management of Stuttering Priapism and Sleep-Related Painful Erection. J. Sex. Med..

[49] Lowe F.C., Jarow J.P. (1993). Placebo-controlled study of oral terbutaline and pseudoephedrine in management of prostaglandin E1-induced prolonged erections. Urology.

[50] Migliorini F., Porcaro A.B., Baldassarre R., Artibani W. (2016). Idiopathic stuttering priapism treated with salbutamol orally: A case report. Andrologia.

[51] Perimenis P., Athanasopoulos A., Papathanasopoulos P., Barbalias G. (2004). Gabapentin in the management of the recurrent, refractory, idiopathic priapism. Int. J. Impot. Res..

[52] Burnett A.L., Bivalacqua T.J., Champion H.C., Musicki B. (2006). Long-term oral phosphodiesterase 5 inhibitor therapy alleviates recurrent priapism. Urology.

[53] Nardozza A.J., Cabrini M.R. (2017). Daily use of phosphodiesterase type 5 inhibitors as prevention for recurrent priapism. Rev. Assoc. Med. Bras. (1992).

[54] Massenio P., D’Altilia N., Sanguedolce F., Carrieri G., Cormio L. (2018). Daily tadalafil for the chronic phase of stuttering priapism: A case report. BMC Urol..

